# Comparisons of Adherence to Antiretroviral Therapy in a High- Risk Population in China: A Systematic Review and Meta-Analysis

**DOI:** 10.1371/journal.pone.0146659

**Published:** 2016-01-12

**Authors:** Zhou Huan, Wang Fuzhi, Liu Lu, Zhang Min, Chen Xingzhi, Jin Shiyang

**Affiliations:** 1 The First Affiliated Hospital of Bengbu Medical College, Bengbu, Anhui, 233000, P. R. China; 2 Health Management Department, Bengbu Medical College, Bengbu, Anhui, 233000, P. R. China; University of Rome Tor Vergata, ITALY

## Abstract

**Background:**

Reports on antiretroviral therapy (ART) adherence are scare in China; we performed this meta-analysis to estimate ART adherence rates in different populations at high risk for HIV transmission in China.

**Methods:**

We searched PubMed, Chinese Biomedical Literature Database (Chinese), China National Knowledge Infrastructure (Chinese), and Wanfang (Chinese) to identify studies published from January 1985 to May 2015. We used random-effects meta-analysis to calculate weighted mean estimates across studies and 95% CIs. Data were pooled with proportions transformed prior to pooling using the Freeman–Tukey double arcsine transformation and then back transformed to the original scale. We calculated the I^2^ (and its 95% confidence intervals) and tau^2^ to assess between-study heterogeneity.

**Results:**

We identified 36 eligible articles, including 6885 HIV-positive individuals, reporting ART adherence. Pooled analysis produced an estimate of 77.61% (95% CI = 71.63–83.08) of patients with HIV with adequate adherence; however, high heterogeneity was observed between studies (I^2^ = 96.60%, 95%CI = 96.00%-97.20%; tau^2^ = 0.16). Three studies, which included 149 old HIV-infected patients, reported the highest ART adequate adherence rate (89.39%, 95% CI = 72.01–99.26) with high heterogeneity between the studies (I^2^ = 86.20%, 95%CI = 60.00–95.20%; tau^2^ = 0.13). While, only two studies, which included 143 heterosexual transmission group (HTG) patients, reported the lowest ART adequate adherence rate (51.55%, 95% CI = 41.33–61.71) with low heterogeneity between the studies (I^2^ = 31.3%, tau^2^ = 0.007). In the multivariable meta-regression model, high-risk populations was the main factor explaining heterogeneity (variance explained 28.14%).

**Conclusions:**

ART adherence in some high-risk populations (e.g., heterosexual transmission group) is below the recommended levels for maintaining virologic suppression. It is crucial to develop comprehensive intervention strategies to promote ART adherence in high-risk populations and effectively prevent the spread of HIV/AIDS in China.

## Introduction

Latest data shows an estimated 35·3 million people lived with HIV, of which 9·7 million people in low-income and middle-income countries were receiving antiretroviral therapy (ART) in 2012. Breakthroughs in the “treatment as prevention” strategy, e.g. using ART in pregnant women, people with HIV or with pre-exposure prophylaxis, have resulted in significant reduction in HIV transmission rate, and thus are very important for public health[1]. HIV ART with adequate adherence is conventionally the most effective prevention strategy, known as “treatment as prevention”, due to its significant association with a reduced HIV transmission rate. Furthermore, ART can help HIV-infected patients restore immune function, prolonging life and improving the quality of life[2]. Adequate adherence to ART plays an important role in sustaining viral suppression, preventing the development of disease and decreasing the mortality rate. Drug resistance quickly occurs during the therapeutic process due to poor adherence, which may reduce treatment efficacy, promote disease progression, and increase the risk of transmission [3]. However, many HIV-infected individuals do not achieve or maintain high levels of adherence. Globally, nearly 40% of HIV-infected patients did not maintain an adherence rate higher than 90% in 2011[4].

China is the most HIV-affected region in Asia. HIV epidemics in China tend to be concentrated among members of groups at high risk of acquiring or spreading HIV[5]. However, no systematic review of the ART adherence in China has been performed. Further, access to antiretroviral therapy and adherence in different populations may vary, and no systematic review of comparisons and variations in ART adherence between different populations has been performed.

We performed this meta-analysis to provide a pooled proportion of HIV-infected patients with adequate ART adherence in different high-risk populations to prevent the spread of HIV/AIDS in China. We also provide comparisons of ART adherence between these important populations.

## Methods

### Search Strategy and Selection Criteria

We searched PubMed, Chinese Biomedical Literature Database (CBM), China National Knowledge Infrastructure (CNKI), and Wanfang (Chinese) to identify any study in each database published from January 1985 to May 2015. The following detail search strategy was used in PubMed: (HIV or “human immune*” or “acquired immunodeficiency syndrome”) and (“China” or “Chinese”) and (“adherence” or “compliance” or “pill counts”) and (“ART” OR ‘‘HAART” OR ‘‘antiretroviral*”). The same search strategies were used for each database. No language restriction was placed on the search process. In addition, we also screened the references of the retrieved paper. If the eligible papers were not obtained, we would contact the corresponding author of the paper for detailed information via e-mail.

Only descriptive epidemiological studies that reported ART adherence in HIV-positive specific groups, such as pregnant women, children, former commercial and plasma donors (FCPD), injection drug users (IDUs), men who have sex with men (MSM), heterosexual transmission group (HTG), were eligible. We excluded qualitative studies, literature reviews, case studies, cost-effectiveness studies, meetings, discussions, editorials, research overviews, book reviews, letters, and news articles. We excluded studies that did not provide useable data, had fewer than 30 participants and were conducted in countries outside China.

### Study Selection and Data Extraction

**Fig 1:**
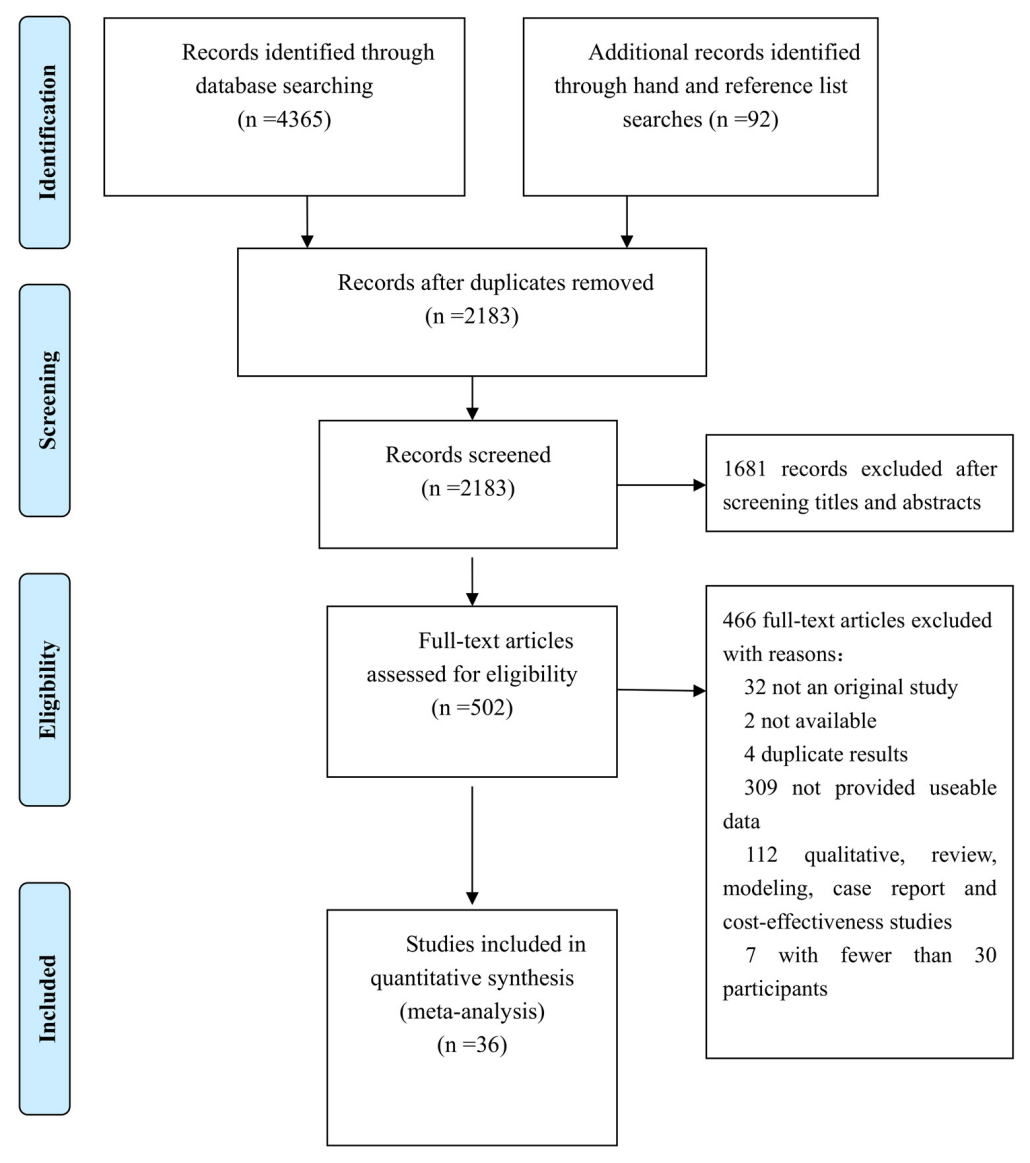
PRISMA 2009 flow diagram literature search and study selection. PRISMA diagram showing the different steps of systematic review, starting from literature search to study selection and exclusion.

Two authors (ZH and WFZ) independently screened the titles and abstracts of all of the identified studies. For each study, one reviewer (LL) extracted the data and a second reviewer (ZM) checked the accuracy. Disagreements would be discussed and resolved by a third investigator (CXZ) if these two investigators could not reach a consensus. For the 36 studies included here, there were no disagreements between the first reviewer (LL) and the second reviewer (ZM). Fig 1 shows the flowchart for selecting articles [6–41].

We developed a data abstraction form and extracted the following data from the eligible studies: studies characteristics (authors, years, design, and study regions), study population (age, sex, and sample size), ART regimen, adherence threshold (e.g., 90%, 95%, 100%), methods of outcomes measurement, and adherence assessor. The population was divided into pregnant women, children, FCPD, IDUs, FSP of HIV-positive individuals, MSM, and TB/HIV co-infected people. We also obtained data from the website of the Chinese administrative region; these data were used to categorize regions (eastern China, south-central China, north China, northwest China, southwestern China, and northeast China) and the gross domestic product in $US per individual in 2013 (low income (<$5520), low-middle income ($5635–6750), high-middle income ($6892–9961), and high income (>$10,915)[42,43].

### Quality Assessment

We used the Agency of Healthcare Research and Quality (AHRQ) assessment to assess the quality of the eligible literature. The AHRQ is an 11-item questionnaire with three-options for each item, and the total score of the AHRQ is 11. Article quality was assessed as low (from 0 to 3), moderate (from 4 to 7), or high (from 8 to 11)[44].

### Statistical Analysis

Adequate adherence was defined as the proportion of people who reported≥90% adherence to ART, which was referred in each study as the effect size index (ES). Random-effects meta-analysis was used to calculate weighted mean estimates across studies and 95% CIs, because random-effects models are more conservative and provide better estimates with wider confidence[45]. Data were pooled with proportions transformed prior to pooling using the Freeman–Tukey double arcsine transformation and then back transformed to the original scale[46]. We calculated the I^2^ (and its 95% confidence intervals) and tau^2^ to assess between-study heterogeneity, and H^2^ (and its 95% confidence intervals) to assess within-study heterogeneity [47,48].

Subgroup analyses and meta-regression analyses were used to explore potential heterogeneity in the following categories: study regions (eastern China, south China, south-central China, north China, northwest China, southwestern China, and northeast China), publication year, study design (observational or interventional study), adherence threshold (90–99% and 100%),), measure of adherence (single self-reported, combined method of two or more single measure methods including self-reported, pill count, checking medical records, and clinic staff monitoring in the whole study process)), sample size (≤100 and >100), and study quality score (<8 and ≥8). The subgroup analyses in MSM, FCPD, and HTG of HIV-infected patients were not performed because that the number of studies (n≤2) in these subgroup were too small.

To establish the robustness of the outcome by sensitivity analyses, we applied a fixed effects model, excluded studies with a low number of participants[49], and excluded studies with a low quality score[19]. A funnel plot was used to explore publication bias. Funnel-plot asymmetry was further assessed by a Begg’s test and Egger’s linear regression test[50]. We performed all analyses using Stata software (version 13.0) and R software (version 2-15-2).

## Results

### Characteristics of Included Studies

We identified 4365 papers from database searches and 92 papers through Internet, hand, and reference list searches. During the abstract screening, 3955 papers were excluded, leaving 502 full-text papers to be assessed for eligibility. Finally, we included 36 papers in this meta-analysis (Fig 1).

Table 1 notes the characteristics of the 36 eligible studies. Most studies were conducted in south-central China (n = 17, 47.22%), followed by south-western China (n = 8, 22.22%), northwest China (n = 4, 11.11%), and trans-regional cities (n = 4, 11.11%). Adherence was most commonly reported in studies of pregnant women (n = 14, 38.89%), followed by IDU (n = 9, 25%), children (n = 4, 11.11%) and elderly patients (n = 3, 8.33%). Most studies (n = 28, 77.78%) were observational, and 8 (22.22%) were interventional studies. 10 (27.78%) studies used a self-reported adherence rate, whereas 26 (72.22%) used combined method of two or more single measure methods including self-reported, pill count, checking medical records, and clinic staff monitoring in the whole study process.

**Table 1:** Characteristics of Studies Included in the Meta-Analysis. Note: & Quality score assessed by Agency of Healthcare Research and Quality (AHRQ); $ Income level is divided to high income (>10915), high-middle income (6892–9961), low-middle income, low income (<5520) according to GDP (in $US per head); FCPD = former commercial and plasma donors; IDU = injection drug users; MSM = men who have sex with men; HTG = heterosexual transmission group; OBS = observational study; INT = interventional study; cART = combined antiretroviral therapy; sdNVP = single-dose nevirapine; AZT = zidovudine; D4T = stavudine; DDI = dideoxyinosine; NVP = nevirapine; EFV = efavirenz; 3TC = Lamivudine; LPV/r = Lopinavir/ritonavir; TDF = tenofovir disoproxil fumarate.

Study(years)	study years	No of participants	Study design	Region	Income level^$^	cART regimens	Adherence Assessor	Adherence (%);Threshold for Measurement	Scores of study quality^&^
Wang YZ (2008)	2005–2006	122 pregnant women	OBS	Southwestern China	Low income	AZT+NVP; sdNVP	Patient or physician	100%; doses taken from 28 weeks of gestation	8
Wang YZ (2008)	2005–2007	158 pregnant women	OBS	Southwestern China	Low income	D4T+3TC+NVP/EFV;AZT+NVP; sdNVP	Patient or physician	100%; doses taken from 28 weeks of gestation	8
Pang J(2009)	2005–2008	110 pregnant women	OBS	South-central China	Low income	Three drugs or two drugs	Clinic staff	100%; doses taken from 28 weeks of gestation	5
Wang YZ (2008)	2005–2007	167 pregnant women	OBS	Southwestern China	Low income	D4T+3TC+NVP/EFV;AZT+NVP; sdNVP	Medical record	100%; doses taken from 28 weeks of gestation	8
Wang LH(2009)	2004–2008	312 pregnant women	OBS	Trans-regional	Low income	standard therapeutic regimen for pregnant women	Clinic staff	100%; doses taken from 28 weeks of gestation	7
Wang Q(2011)	2005–2006	1490 pregnant women	OBS	Trans-regional	Low income	D4T+3TC+NVP/EFV;AZT+NVP; sdNVP	Patient or physician	100%; doses taken from antenatal to postpartum	8
Gui XZ(2012)	2006–2010	208 pregnant women	OBS	South-central China	Low income	AZT+3TC+NVP/EFV	Self-reported	100%; doses taken from antenatal to postpartum	6
Hu HM(2012)	2010–2011	678 pregnant women	INT	Southwestern China	Low income	AZT+3TC+LPV/r;AZT+3TC+EFV/NVP	Patient or physician	100%; doses taken from antenatal to postpartum	8
Li AJ (2012)	2006–2008	158 pregnant women	OBS	Southwestern China	Low income	AZT+3TC+NVP/EFV; AZT+NVP; sdNVP	Medical record	95%; based on past 1 month	6
Chen SP(2013)	2012	30 pregnant women	INT	South-central China	High-middle income	No report	Self-reported	95; doses taken in past 1 months	5
AILIKA.Shawuli(2013)	2010–2012	1303 pregnant women	OBS	Northwest China	Low-middle income	AZT; 3TC+NVP; sdNVP; AZT+3TC; AZT+3TC+LPV/r; AZT+3TC+EFV/NVP	Patient and physician	100%; doses taken from antenatal to postpartum	6
Wang Q(2013)	2005–2008	1414 pregnant women	OBS	Trans-regional	Low income	Standard therapeutic regimen for pregnant women and newborn	Medical record and patient report	100%; doses taken from antenatal to postpartum	8
Wu HP(2013)	2009–2011	314 pregnant women	OBS	Southwestern China	Low income	Standard therapeutic regimen for pregnant women	Patient and physician	100%; doses taken from antenatal to postpartum	7
Li ZJ(2014)	2005–2013	69 pregnant women	OBS	Southwestern China	Low income	AZT+3TC+LPV/r	Patient and physician	100%; doses taken from antenatal to postpartum	4
Yu RH(2007)	2006	72 FCPD	OBS	Eastern China	Low income	D4T+3TC+NVP/ATV;D4T+DDI+NVP;3TC+DDI+EFV	Self-reported	95%; No. of pills taken/pills prescribed in past four days	8
Yuan Y(2012)	2003–2009	606 FCPD	OBS	South-central China	Low income	AZT/D4T+DDI+NVP/EFV;AZT/D4T+3TC+NVP/EFV	Patient or physician	90%; doses taken in past 1 month	6
Lu J(2008)	2006	62 IDUs	OBS	Northwest China	Low-middle income	D4T+3TC+NVP/EFV;AZT+3TC+NVP	Self-reported	95%; based on past 1 week	8
Wang HH(2008)	2007	111 IDUs	OBS	South-central China	Low income	AZT+3TC+NVP/EFV;	Self-reported	90%; No. of the doses taken/No. of doses prescribed in past 1 month	7
Zhou J (2008)	2007–2008	116 IDUs	INT	South-central China	Low-middle income	AZT+3TC+NVP/EFV;	Clinic staff and pill count	>95%; No. of pills taken/No. of pills prescribed over prior 1 month	9
Lu J(2008)	2007–2008	180 IDUs	OBS	Trans-regional	Low-middle income	D4T+3TC+NVP/EFV;AZT+3TC+NVP/EFV	Self-reported	95%; No. of doses recorded/total No. of doses prescribed in the past 1 week	9
Yang CB(2010)	2007–2009	102 IDUs	OBS	Southwestern China	Low income	D4T+3TC+NVP;AZT+3TC+NVP/EFV	Patient or physician	100%; based on past 12 months	6
Su FQ(2011)	2007	60 IDUs	INT	South-central China	Low income	No report	Self-reported	100%; doses taken in past 1 month	4
Lu J(2013)	2012	180 IDUs	OBS	Northwest China	Low-middle income	No report	Pill count	95%; No. of pills taken/No. of pills prescribed	7
Qin YL(2013)	2009–2011	73 IDUs	INT	South-central China	High-middle income	AZT+3TC+NVP/EFV; D4T+3TC+NVP/EFV;	Self-reported	100%; doses taken/ doses prescribed in the past 1 week	6
Ye HL(2013)	2010	60 IDUs	OBS	South-central China	Low income	AZT+3TC+NVP/EFV/LPV/r; D4T+3TC+NVP/EFV;TDF+3TC+LPV/r	Clinic staff	100%; doses taken in past 6 months	6
Deng MH(2012)	2010	72 elderly patients	INT	South-central China	Low income	No report	Patient or physician	100; doses taken in past 1 month	5
Liu JR(2013)	2012	63 elderly patients	OBS	South-central China	Low income	No report	Patient and physician	100%; doses taken in past 3 months	4
Lu MR(2014)	2013	100 elderly patients	INT	South-central China	Low income	No report	Clinic staff and pill count	100%; doses taken/ doses prescribed in the past 6 months	4
Zhou YF(2012)	2009–2012	200 MSM	INT	South-central China	Low-middle income	No report	Self-reported	100%; doses taken	8
Qiao JK(2014)	2010–013	200 MSM	OBS	Northern China	Low income	AZT/D4T+3TC+NVP/EFV	Clinic staff and pill count	95%; No. of pills taken/No. of pills prescribed	7
Liu AW(2007)	No report	40 children	OBS	Eastern China	Low income	D4T+3TC+NVP/EFV;AZT+3TC+NVP/EFV;	Clinic staff	100%; based on past 1 month	6
Chang YL(2009)	2007–2009	46 children	OBS	Northwest China	Low-middle income	D4T+3TC+NVP/EFV;AZT+3TC+NVP/EFV	Patient or physician	100%; based on past 3 months	5
Liu X (2011)	2009	87 children	OBS	South-central China	Low income	AZT/D4T+3TC+EFV;AZT/D4T+3TC+LPV/r;ABC+3TC+LPV/r	Plasma drug monitoring	100%; plasma concentration of drugs higher than 1000ng/ml in the past 3 visits	8
Wei XB(2012)	2005–2011	49 children	OBS	South-central China	Low income	Standard therapeutic regimen for children	Pill count and self-reported	100%; No. of pills taken/No. of pills prescribed	4
Dong XB(2014)	No report	93 HTG	OBS	South-central China	Low-middle income	No report	Medical record	100%; No. of pills taken/No. of pills prescribed	4
Lee SS (2007)	No report	50 HTG	OBS	South-central China	High income	No report	self-reported	100%; No. of pills taken/No. of pills prescribed	7

Table 1 presents the study quality score assessed using the AHRQ; 12 (33.33%) were scored as high quality, and 24 (66.67%) were scored as moderate quality.

### Overall Adherence to Antiretroviral Therapy

**Fig 2:**
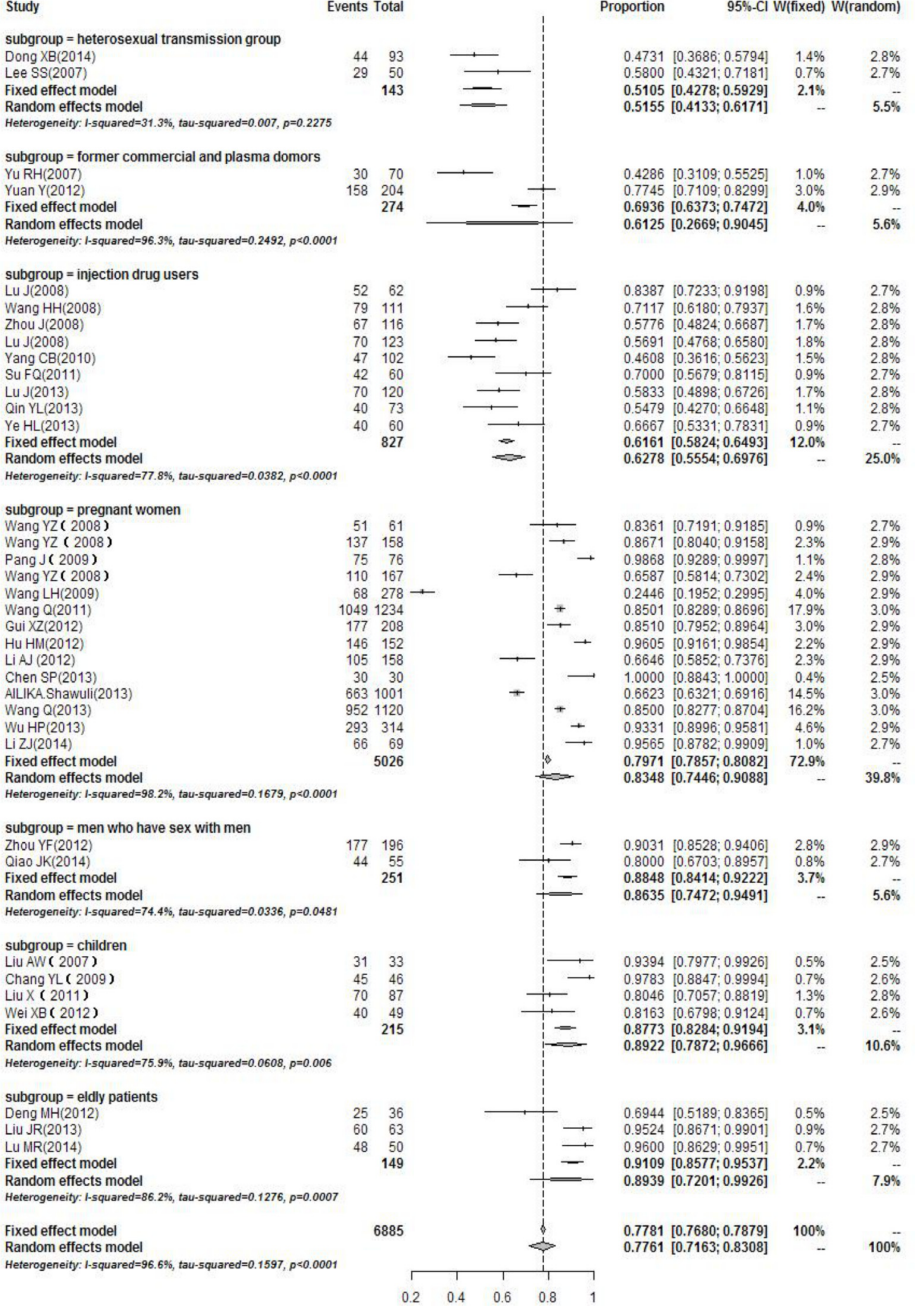
Meta-analyses of adequate adherence to antiretroviral therapy in special population groups in china. Adequate adherence was defined as the proportion of people who reported≥90% adherence to ART, which was estimated in each study as the effect size index (ES).

Thirty-six studies, which included 6885 HIV-positive individuals, reported ART adherence (Fig 2). Approximately 77.61% (95% CI = 71.63–83.08) of HIV patients had a high level of adherence; however, high levels of heterogeneity were observed between studies (I^2^ = 96.6%, 95%CI = 96%-97.2%;tau^2^ = 0.1597;H = 5.44, 95%CI = 4.98–5.95).

### ART Adherence Rate by Different Subgroups

The ART adequate adherence rate varied widely between different high-risk populations (Fig 2). Three studies, which included 149 old HIV-infected patients, reported the highest ART adequate adherence rate (89.39%, 95% CI = 72.01–99.26) with high heterogeneity between the studies (I^2^ = 86.2%, 95%CI = 60–95.2%; tau^2^ = 0.128). While, only two studies, which included 143 heterosexual transmission patients, reported the lowest ART adequate adherence rate (51.55%, 95% CI = 41.33–61.71) with low heterogeneity between the studies (I^2^ = 31.3%, tau^2^ = 0.007).

A total of 14 studies reported ART adequate adherence rate in the subgroup of pregnant women, and the rate was 83.48% (95% CI = 74.72–94.91); however, high heterogeneity was observed between the studies (I^2^ = 98.20%, 95%CI = 97.7–98.6; tau^2^ = 0.168). The subgroup analyses in pregnant women were also performed, and we found that the adequate adherence rate in pregnant women continually increased from 79.18% (95% CI = 63.80–92.2) in 2008 to 95.65% (95% CI = 89.27–99.45) in 2014 (Table 2). The region with the highest adherence rate in pregnant women was south-central China (96.05%, 95% CI = 83.13–100), followed by south-western China (85.68%, 95% CI = 74.18–93.93) and northwest China (66.23%, 95% CI = 62.27–69.13). The studies with small sample size reported higher adherence rate (95.89%, 95% CI = 87.93–99.94%) than that in studies with large sample size (77.45%, 95% CI = 66.06–87.09%) in pregnant women (Table 2).

**Table 2:** Subgroup Analyses of Adherence (95%CI) to Antiretroviral Therapy in Special Population Groups in China.^&^ Note: & = Number of studies (k≤2) too small to test for small study effects. $ = combined method of two or more single measure methods including self-reported, pill count, checking medical records, and clinic staff monitoring in the whole study process.

Subgroup		No. of studies	Proportion, 95%CI(100%)	Heterogeneity		
				I^2^, 95%CI(%)	H, 95%CI	tau^2^
Pregnant women	Publication year					
	2008	3	79.18(63.80 to 91.00)	90.7(75.5 to 96.4)	3.27 (2.02 to 5.3)	0.081
	2009	2	68.60 (27.10 to 100)	99.5	14.17	1.664
	2011	1	85.01(82.96 to 86.95)	-	-	-
	2012	3	84.43(65.34 to 96.84)	96.2(92 to 98.2)	5.14(3.53 to 7.48)	0.148
	2013	4	88.11(74.41 to 97.15)	98.4(97.4 to 99)	7.84(6.18 to 9.94)	0.12
	2014	1	95.65(89.27 to 99.45)	-	-	-
	Regions					
	Northwest China	1	66.23 (63.27 to 69.13)	-	-	-
	South-central China	3	96.05(83.13 to 100)	99.5 (99.3 to 99.7)	14.6 (12.06 to 17.68)	0.274
	Southwestern China	7	85.68(74.81 to 93.93)	94.9 (91.8 to 96.9)	4.44 (3.49 to 5.66)	0.126
	Trans-reginoal	3	66.77(37.46 to 90.32)	90.7 (75.6 to 96.5)	3.28(2.02 to 5.31)	0.125
	Sample size					
	≤100	4	95.89(87.93 to 99.94)	78.9(43.6 to 92.1)	2.18(1.33 to 3.56)	0.065
	>100	10	77.45(66.06 to 87.09)	98.6 (98.2 to 98.9)	8.49(7.45 to 9.69)	0.163
	Adherence measure					
	Single self-reported	2	94.40(72.45 to 100)	90.1	3.18	0.171
	Combined methods^$^	12	81.42(71.16 to 89.88)	98.4(98 to 98.8)	7.97(7.04 to 9.02)	0.174
	Quality score of study quality					
	<8	8	82.86(64.92 to 95.38)	98.7(98.2 to 99)	8.71(7.52 to 10.08)	0.338
	≥8	6	84.74(79.13 to 89.62	91.2 (83.7 to 95.3)	3.38 (2.48 to 4.61)	0.027
Injection drug users	Publication year					
	2008	4	67.47(55.36 to 78.52)	84.4(60.9 to 93.8)	2.53(1.6 to 4)	0.053
	2010	1	46.08(36.47 to 55.84)	-	-	-
	2011	1	70(57.72 to 81.02)	-	-	-
	2013	3	59.34(53.17 to 65.36)	0(0 to 89.5)	1(1 to 3.09)	0
	Regions					
	Northwest China	2	71.61(44.38 to 92.40)	92.4	3.62	0.147
	South-central China	5	64.05(57.24 to 70.60)	50.5(0 to 81.8)	1.42(1 2.35)	0.012
	Southwestern China	1	46.08(36.47 to 55.84)	-	-	-
	Trans-reginoal	1	56.91(48.04 to 65.56)	-	-	-
	Sample size					
	≤100	4	69.23(56.37 TO 80.77)	78.3(41.4 to 91.9)	2.14 (1.31 to 3.52)	0.056
	>100	5	58.22(50.49 to 65.75)	71.7(28.7 to 88.8)	1.88(1.18 to 2.99)	0.022
	Adherence threshold					
	90–99%	4	67.59(55.65 to 78.49)	84.1(60 to 93.7)	2.51(1.58 to 3.98)	0.052
	100%	5	58.56(50.20 to 66.70)	65.2(9 to 86.7)	1.7(1.05 to 2.74)	0.023
	Adherence measure					
	Single self-reported	5	67.54(56.98 to 77.28)	80.2(53.3 to 91.6)	2.24(1.46 to 3.44)	0.048
	Combined methods^$^	4	56.75(49.01 to 64.33)	58.1 (0 to 86.1)	1.55(1 to 2.68)	0.014
	Quality score of study quality					
	<8	3	66.36 (49.73 to 81.17)	72.9(37.8 to 88.2)	1.92(1.27 to 2.91)	0.031
	≥8	6	61.09(52.78 to 69.10)	88.2(67.1 to 95.8)	2.91(1.74 to 4.86)	0.077
Children	Publication year					
	2007	1	93.94(82.59 to 99.89)	-	-	-
	2009	1	97.83(90.90 to 1)	-	-	-
	2011	1	80.46(71.4 to 88.19)	-	-	-
	2012	1	81.63(69.41 to 91.40)	-	-	-
	Regions					
	Eastern China	1	93.94(82.59 to 99.89)	-	-	-
	Northwest China	1	97.83(90.90 to 1)	-	-	-
	South-central China	2	80.92(73.77 to 87.20)	75.9(33.6 to 91.2)	2.04(1.23 to 3.38)	0.061
	Quality score of study quality					
	<8	3	92.11(80.28 to 99.15)	72.9(9 to 92)	1.92(1.05 tp 3.53)	0.063
	≥8	1	80.46(71.40 to 88.19)	-	-	-
Elderly patients	Publication year					
	2012	1	69.44(53.28 to 83.58)	-	-	-
	2013	1	95.24(88.28 to 99.39)	-	-	-
	2014	1	96(88.32 to 99.93)	-	-	-

A subgroup analysis of ART adherence rate in IDUs was also performed (Table 2). In IDUs, two studies reported the highest adherence rate in northwest China (71.61%, 95% CI: 44.38–92.40) with high heterogeneity between the studies (I^2^ = 92.4%, tau^2^ = 0.147); while only one studies reported the adherence rate in southwest China, which reported the lowest adherence rate (46.08%, 95% CI: 36.47–55.84).

Analyses of ART adherence rate in elderly and pediatric patient subgroups were also performed. However, no significant variations were observed between the subgroups in publication year, regions, and study quality (Table 2). Moreover, the subgroup analyses in MSM, FCPD, and HTG were not performed because only two studies were available for these specific groups.

### Meta-Regression Analyses

The meta-regression for ART adherence is shown in Table 3. In the univariate meta-regression model, high-risk populations, sample size, and study quality score were associated with ART adherence rate. In the multivariable model, high-risk populations was the main factors explaining heterogeneity (variance explained 28.14%). Sample size and study quality score did not retain significance.

**Table 3:** Results of Meta-regression for Adherence to Antiretroviral Therapy in China. Note: $, combined method of two or more single measure methods including self-reported, pill count, checking medical records, and clinic staff monitoring in the whole study process. OBS = observational study; INT = interventional study;

Covariate	No. of studies	Univariate analyses			Multivariable analyses		
		Coefficient (95% CI)	P value	Variance	Coefficient (95% CI)	P value	Variance
				explained (%)			explained (%)
Population (HTG is reference)			0.028	24.46		0.032	28.14
Former commercial and plasma donors	2	0.09(-0.26 to 0.43)	0.618		0.11(-0.26 to 0.48)	0.551	
Injection drug users	9	0.10(-0.17 to 0.38)	0.439		0.13(-0.16 to 0.41)	0.367	
Pregnant women	14	0.29(0.02 to 0.55)	0.034		0.30(0.03 to 0.72)	0.029	
Men who have sex with men	2	0.33(-0.01 to 0.67)	0.06		0.36(0.01 to 0.72)	0.047	
Children	4	0.36(0.06 to 0.66)	0.019		0.36(0.02 to 0.71)	0.038	
Elderly patients	3	0.35(0.04 to 0.67)	0.03		0.32(0.01 to 0.67)	0.041	
Regions (trans-regional is reference)			0.791	-8.13			
Eastern China	2	0.06(-0.29 to 0.41)	0.722				
Northwest China	4	0.13(-0.15 to .41)	0.466				
South-central China	17	0.14(-0.08/ to 0.36)	0.206				
Southwestern China	8	0.17(-0.08 to 0.41)	0.173				
Northern China	1	0.17(-0.28 to 0.62)	0.447				
Study design (INT vs reference)	36	0.06(-0.10 to 0.21)	0.464	-1.11			
Income level	36	-0.05(-0.15 to 0.06)	0.371	-0.78			
Sample size (≤100 vs >100)	36	-0.14(-0.25 to -0.02)	0.027	10.77	0.01(-0.20 to 0.21)	0.958	
Quality score of study quality	36	-0.03(-0.07 to 0.00002)	0.050	7.8	-0.02(-0.07 to 0.02)	0.328	
Year of publication	36	0.02(-0.001 to 0.05)	0.064	6.68			
Adherence Assessor(single self-reported vs combined methods$)	36	0.05(-0.03 to 0.17)	0.197	2.35			
Threshold for Measurement (%)	36	0.04(-0.05 to 0.12)	0.355	-0.17			

### Sensitivity Analyses

We performed sensitivity analyses of the ART adherence rate by applying a fixed effects model, and we found similar adherence rate between random-effect mode and fixed-effect mode in the overall analysis and the subgroup analyses (Fig 2). Moreover, excluded studies with a low number of participants, and excluded studies with a low quality score both produced similar adherence rate.

### Evaluation of Publication Bias

**Fig 3:**
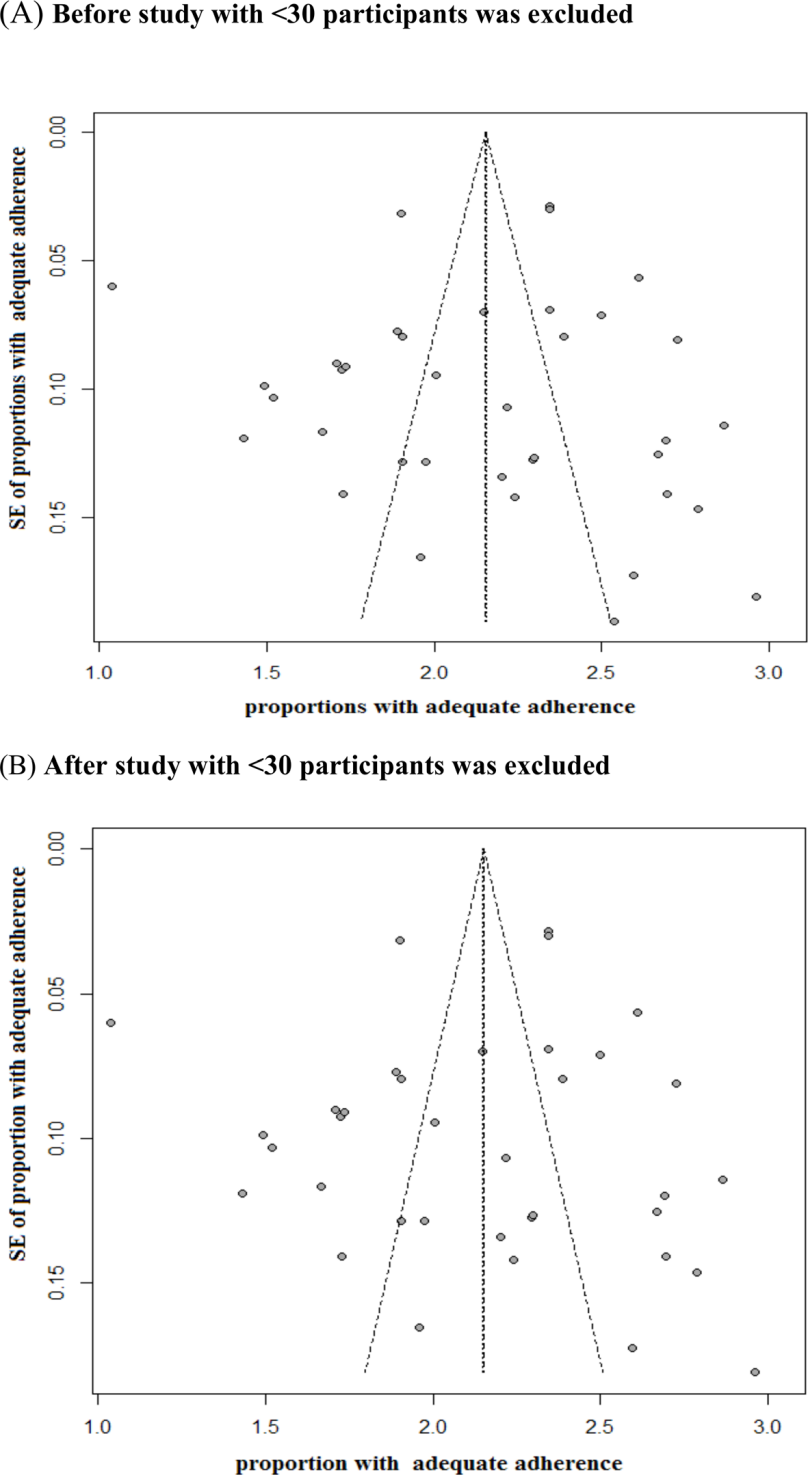
Funnel plot for adequate adherence to antiretroviral therapy in China. Panel A: Funnel Plot before study with <30 participants was excluded, Freeman-Tukey Double arcsine transformation was used, Egger’S Linear Regression Test (t = -0.282, P = 0.779); Panel B: Funnel Plot after study with <30 participants was excluded, Egger’S Linear Regression Test (t = -0.365, P = 0.717).

We produced funnel plots and visually examined these plots for signs of asymmetry. We generated funnel plots to assess publication bias of the ART adherence rate. For overall ART adherence rate, the asymmetry observed in the funnel plots was minimal (Fig 3). We also assessed funnel plot asymmetry using the Egger’s linear regression test. No publication bias was observed in the adherence estimate across different specific populations (P>0.05) (Table 4).

**Table 4:** Heterogeneity test and Egger’s linear regression test to measure the funnel plot asymmetric.^&^ Note: &, Number of studies (k≤2) too small to test for small study effects

Population	No. of studies	Heterogeneity			Egg's test	
		I^2^, 95%CI(%)	H, 95%CI	tau^2^	t	P
Pregnant women	14	98.2(97.7 to 98.6)	7.45 (6.61 to 8.39)	0.168	0.43	0.668
Men who have sex with men	2	74.4	1.98	0.034	-3.14	-
Injection drug users	9	77.8(57.9 to 88.3)	2.12(1.54 to 2.92)	0.038	-0.32	0.962
Former commercial and plasma donors	2	96.3	5.21	0.249	-11.56	-
Heterosexual transmission group	2	31.3	1.21	0.007	-3.08	-
Elderly patients	3	86.2(60 to 95.2)	2.69(1.58 to 4.58)	0.128	-4.06	0.107
Children	4	75.9(33.6 to 91.2)	2.04(1.23 to 3.38)	0.061	-5.01	0.144

## Discussion

To our knowledge, this is the first meta-analysis to summarize and compare pooled ART adherence between different high-risk populations in China. We found that approximately 76.6% of HIV-infected individuals at high risk of transmitting HIV have adequate ART adherence. Significant differences in adherence rate were observed between different populations, and the highest ART adherence rate was observed in elderly patients, followed by children, MSM, pregnant women, IDUs, FCPD, and HTG. The adequate ART adherence rate in China was higher than that in India (70%), Spain (50%), and North American countries (57%) and similar to African countries[51–53]. Our findings indicated that reaching and maintaining high levels of ART adherence remain major issues in China, especially in some high-risk populations with lower adherence.

The use of ART in decreasing mother-to-child transmission of HIV has played an important role in global HIV/AIDS prevention and control. A recent meta-analysis observed a pooled estimate of 62.0% of pregnant women who had more than 80% ART adherence in high-income countries[54]. Our review observed a pooled estimate of nearly 80% of Chinese pregnant women who had 100% ART adherence, which might be due to the Chinese government investing in integrated prevention of mother-to-child transmission (PMTCT). The Chinese government has increased the investment for integrated PMTCT from US$0.9 million in 2003 to US$136.8 million in 2010, resulting in a remarkable increase of ART in HIV-infected pregnant[55]. In China, PMTCT services were integrated with antenatal and perinatal care, and HIV-infected pregnant women were treated with antiviral drugs when they attended antenatal/perinatal care service. The integration of these health services in terms of HIV prevention may contribute to the maintenance of a high level of ART adherence.

HIV epidemics in China continue to be concentrated among FSWs, IDUs, and MSM, not in the general population; therefore, maintaining high levels of ART adherence in these high-risk populations may be very important for preventing and reducing HIV transmission in China[1]. Some previous reviews have observed that 76% of FSWs worldwide and 72% of IDUs in low- and middle-income countries have adequate ART adherence[2,56]. Our meta-analysis estimated that 62.78% of IDUs had adequate ART adherence in China, which is a very low adherence level compared with that of other low- and middle-income countries. In China, many barriers prevent IDUs from maintaining ART high adherence. For example, poverty can affect adherence by limiting travel to centres for medication, and stigma may also prevent patients from utilizing available free ART services. Several programs, such as needle and syringe exchange programs, have now begun to integrate treatment for opiate addiction into current free ART programs for IDUs in China, which can promote ART adherence. Regarding MSM, no review has assessed ART adherence. Although we observed an encouraging estimate of ART adherence in MSM, this estimate should be interpreted with caution due to the small number of studies. Although exposure among MSM has become the dominant route of HIV transmission, Chinese MSM tend to have very poor access to HIV prevention services, with only a few national prevention programs targeting this population in China[1]. A very interesting finding in this meta-analysis was that the lowest ART adequate adherence rate was observed in heterosexual transmission group patients. Considerable sexual transmission continues among MSM and high risk heterosexuals in China, therefore we really have to exert more effort and spend more time on the “treatment as prevention” strategy in these high risk populations.

Our study had some limitations. Firstly, a high heterogeneity was observed between studies. Although we performed subgroup analyses according to different high risk populations, publication year, geographical area, sample size, threshold for measurement, adherence assessor and study quality, these factors may be the sources of between-study heterogeneity. However, other unmeasured factors likely affected the detected heterogeneity; unfortunately, we did not obtain sufficient information about these aspects for further analysis. Assuming homogeneity often results in a misleading analysis, since heterogeneity is very likely present but undetected, especially for very small meta-analyses[15]. However, we could not check that the findings stand even if we assume high levels of undetected heterogeneity. Furthermore, not enough studies for a meta-regression, especially for the multiple regression in our meta-analysis. Small meta-analyses tend not to detect existing heterogeneity and we need to be cautious when they observe apparent homogeneity [57]. Secondly, we did not detect publication bias using Egger’s linear regression test in most of our analyses. However, these results should be interpreted with caution because the detect power is limited, particularly for moderate amounts of bias or meta-analyses based on a small number of small studies (<10). The power to detect publication bias increased with increasing numbers of studies, and publication bias tests only relevant if there are >10 studies otherwise underpowered to detect much and tend to lead to conclusions that are not justified. Furthermore, the risk of publication bias in older studies tends to be higher[58], while we did not find that older studies show larger effects. Thirdly, our results could be altered by a selection bias because different number of studies on ART adherence performed in different populations (pregnant women, children), which may impact the results. In 2003, China initiated its first PMTCT programme in eight cities which provided free HIV testing and antiretroviral (ARV) treating and/or prophylaxis through the antenatal care network. This national health service was the most widely covered and completed, with a high follow-up rate. Therefore, studies on HIV prevention and control among pregnant women were easily conducted, and a large number of individual studies related to PMTCT programmes were performed in China. Nevertheless, there was no national health service like antenatal care network to integrate HIV antiretroviral (ARV) treating and/or prophylaxis among other populations such as elderly people, which make it more difficult to conduct studies among these populations. Therefore, selection bias due to the substantially differences in the numbers of studies would be a limitation in this meta-analysis. The last, the single study conducted by Liu X et al. [26] used the ‘blood drug concentration’, one of the most accurate ways of assessing adherence, to assess adherence in this meta-analysis. However, most of the studies included used self-reported method that may introduce information bias or recall bias, which may result in overestimation of the levels of ART adherence.

## Conclusion

In conclusion, our meta-analysis found remarkable variations in adherence rate between different high risk populations, with lower adherence in HTG, FCPD and IDU, and high levels of adherence in elderly patients, children, MSM, and pregnant women. ART adherence in some high-risk population, especially in HTG, was significantly below the recommended level for maintaining virologic suppression. It is crucial to develop comprehensive intervention strategies to promote ART adherence in these high-risk populations and effectively protect against the spread of the HIV/AIDS in China.
